# Receptor-Targeted Fluorescence-Guided Surgery With Low Molecular Weight Agents

**DOI:** 10.3389/fonc.2021.674083

**Published:** 2021-06-30

**Authors:** Servando Hernandez Vargas, Christie Lin, Hop S. Tran Cao, Naruhiko Ikoma, Solmaz AghaAmiri, Sukhen C. Ghosh, Adam J. Uselmann, Ali Azhdarinia

**Affiliations:** ^1^ The Brown Foundation Institute of Molecular Medicine, McGovern Medical School, The University of Texas Health Science Center at Houston, Houston, TX, United States; ^2^ Therapeutics & Pharmacology Program, The University of Texas MD Anderson UTHealth Graduate School of Biomedical Sciences, Houston, TX, United States; ^3^ OnLume, Inc., Madison, WI, United States; ^4^ Department of Surgical Oncology, Division of Surgery, The University of Texas MD Anderson Cancer Center, Houston, TX, United States

**Keywords:** surgical oncology, fluorescence-guided surgery, contrast agents, low molecular weight agents, cancer-targeted agents, receptor targeted imaging

## Abstract

Cancer surgery remains the primary treatment option for most solid tumors and can be curative if all malignant cells are removed. Surgeons have historically relied on visual and tactile cues to maximize tumor resection, but clinical data suggest that relapse occurs partially due to incomplete cancer removal. As a result, the introduction of technologies that enhance the ability to visualize tumors in the operating room represents a pressing need. Such technologies have the potential to revolutionize the surgical standard-of-care by enabling real-time detection of surgical margins, subclinical residual disease, lymph node metastases and synchronous/metachronous tumors. Fluorescence-guided surgery (FGS) in the near-infrared (NIRF) spectrum has shown tremendous promise as an intraoperative imaging modality. An increasing number of clinical studies have demonstrated that tumor-selective FGS agents can improve the predictive value of fluorescence over non-targeted dyes. Whereas NIRF-labeled macromolecules (i.e., antibodies) spearheaded the widespread clinical translation of tumor-selective FGS drugs, peptides and small-molecules are emerging as valuable alternatives. Here, we first review the state-of-the-art of promising low molecular weight agents that are in clinical development for FGS; we then discuss the significance, application and constraints of emerging tumor-selective FGS technologies.

## Introduction

Fluorescence-guided surgery (FGS) is an imaging technique that is uniquely suited to bridge the gap between pre-operative radiologic imaging and post-operative histopathological assessment of cancer. The administration of a fluorescent contrast agent, its localization to sites of interest, and detection by an optical imaging device are the fundamental steps for generating an intraoperative fluorescent image. If effective, a fluorescent agent augments visual feedback to complement tactile guidance in open surgery or overcome the lack of tactile feedback in minimally invasive surgery (MIS), thereby potentially increasing complete tumor resection rates. Indeed, clinical FGS studies have reported improved surgical outcomes and patient benefit with a variety of contrast agents (1–4). The most widely used FGS agent is indocyanine green (ICG; molecular weight [MW] = 776 g/mol; λ_Excitation_/λ_Emission_ [Ex/Em] = 780/820 nm), a non-targeted, water-soluble tricarbocyanine dye and the only FDA-approved near-infrared fluorescence (NIRF) fluorophore. The excellent safety profile and favorable spectral properties of ICG have led to its use in numerous clinical applications such as angiography, tissue perfusion, sentinel lymph node mapping, and tumor imaging (5, 6). Moreover, its hepatic clearance and biliary excretion makes ICG an ideal agent for fluorescence cholangiography, and is increasingly used for safe cholecystectomy (7). Although ICG has been instrumental in demonstrating the utility of FGS (8, 9), there is a critical need to expand FGS applications with agents that possess improved tumor specificity.

Monoclonal antibodies (mAbs) were among the earliest targeted FGS agents to be translated into patients based largely on the repurposing of therapeutic mAbs for imaging (10). Standard bioconjugation techniques, such as N-hydroxysuccinimide (NHS) ester-activated crosslinkers, allowed fluorescent dyes to be reacted with primary amines on lysine residues of mAbs, producing, for example, fluorescent analogs of cetuximab and panitumumab (epidermal growth factor receptor [EGFR] targeting), or bevacizumab (vascular endothelial growth factor receptor [VEGFR] targeting). Clinical studies with these immunoconjugates demonstrated selective receptor binding and feasibility of tumor-specific FGS in several cancers (11–13). However, their large molecular weight produces prolonged serum half-life and slow clearance from non-target tissues. As a result, the time interval between injection and intraoperative imaging with fluorescent mAbs can be as long as one week to generate sufficient tumor contrast. Furthermore, interactions between the Fc region of mAbs and cognate receptors on immune effector cells produce additional background fluorescence that can confound tumor imaging. Low molecular weight (LMW) agents, such as peptides and small molecules, also possess high binding affinity to cancer biomarkers but have more favorable pharmacokinetic (PK) properties than mAb-based agents; for instance, a circulating half-life of a few hours and predominant renal excretion (14). Collectively, these properties have the potential to provide high tumor contrast within a few hours after injection (15, 16). Other key advantages of FGS with LMW agents include tumor visualization at microdose levels, amenability to chemical modification and the absence of immunogenic effects (17, 18).

To further illustrate the clinical potential of LMW agents for FGS, a recent review by Barth and Gibbs listed 39 novel fluorescent agents that are under investigation in clinical trials, 25 of which were classified as peptides or small molecules (19). Several recent review articles have highlighted the clinical development of promising FGS agents (19–22). Here, we focus on LMW agents and their potential to improve intraoperative visualization in surgical oncology. Furthermore, we discuss the significance, application and constraints of emerging tumor-selective FGS technologies.

## Translating LMW Agents Into the Operating Room

The pressing need for improved tumor visualization in the operating room is reflected by the rapidly increasing number of studies under clinical investigation. Although the overarching goals of such studies are clear (i.e., determining safety and efficacy), clinical trial design and endpoint selection are driven by complex factors related to the semi-quantitative and combinatorial nature of FGS (22–24). The lack of standard methods and objective measures of efficacy (25) further complicate the assessment of trial results and trial comparison (26), even when the same drug is used (e.g., in combination with a different imaging device). However, several converging and diverging points during clinical development of LMW agents can be identified. Similarities in early phase trials include determination of an optimal dose and imaging time point along with safety and tolerability, thereby establishing feasibility of the approach. Diverging points are plenty and may be attributed to unique requirements of each clinical application; for instance, anatomical location, spectral properties of the tissue, the identification of margins *vs.* multifocal lesions, etc. (27, 28).

FGS seeks to increase the surgical sensitivity. To date, LMW agents have clinically demonstrated a promising avenue to do so. In this section, we first describe the development of LMW agents that have progressed to clinical studies and meet the specifications outlined in “*Selection Criteria, Search Strategy and Results*.” To contextualize the feasibility of the approach, we then aim to provide a brief description of study design per agent (classified by cancer type), associated endpoints, and emerging applications of FGS that complement current clinical workflows, where appropriate. Finally, in **Tables 1** and **2** we summarize key parameters for discussed studies such as dose, imaging time, device, tumor-to-background ratios (TBRs) and diagnostic accuracy, if available.

**Table 1:** Summary of key parameters in clinical studies with LMW contrast agents.

Agent	Type	Phase	Target	Cancer type	Dose (mg/kg)	Imaging time (h)	Image contrast*	Imaging device	Reference
OTL38	Small molecule	I-III	FRα	Ovarian	0.0125 to 0.2	2-3	3.5-5.4	Artemis (Quest medical imaging); PINPOINT (Novadaq); Iridium	(30, 34)
								(Visionsense)	
				Lung	0.025	3-6	1.0-6.2	Iridium	(35–40)
					(1.48-3.29 mg total)			(Visionsense)	
				Renal	0.025	2	N/A	da Vinci Fluorescence Imaging Vision System (Intuitive Surgical)	(41, 42)
				Gastric adenocarcinoma	0.025	1.5-6	2.6-7.4	VS3 Iridium system	(43)
								(Medtronic)	
				Endometrial carcinoma	0.0125	2-3	2.9-13.0	Artemis (Quest medical imaging)	(44)
				Osteosarcoma	0.025	4	2.9-3.0	Iridium	(45)
								(Visionsense)	
				Pituitary adenoma	0.025	2-4	1.6-3.2	Iridium	(46, 47)
								(Visionsense)	
BLZ-100	Peptide	I-III	Multiple	Gliomas (adult and pediatric)	3-30 mg	3-29	N/A	FLUOBEAM 800 (Fluoptics); SIRIS (Teal Light Surgical)	(48, 49)
(Tozuleristide, Tumor Paint™)							(reported as -, weak or +)		
				Breast carcinoma	6-12 mg	1-26	N/A	SIRIS (Teal Light Surgical)	(50)
ABY-029	Affibody	0-I	EGFR	Soft tissue sarcoma	237 μg	1-3	2.0	Solaris (PerkinElmer)	(51)
					(30 nanomoles)		(n = 1)		
BBN-IRdye800CW	Peptide	I	GRPR	Glioblastoma	1 mg	2-16	3.2-4.9	DPM-III-01 (Zhuhai Dipu Medical Technology)	(52, 53)
cRGD-ZW800-1	Small molecule	I-II	Integrins	Colorectal carcinoma	0.005-0.05	2-18	*In vivo*, 1.1-1.6; *ex vivo*, 1.4-6.2	Olympus Visera Elite II	(54)
								(CLV-S200-IR); Quest	
								Spectrum Platform (Quest Medical Imaging)	
LS301	Peptide	I-II	Annexin A2, others	Breast, liver, pancreas, gastric	-	-	-	-	-

Rows are color coded to facilitate differentiation among agents. *In the image contrast column, we report the average lower and upper limit contrast (e.g., TBR) among all studies in the reference column and may include in situ, ex vivo values.

**Table 2:** Diagnostic accuracy of LMW contrast agents in clinical studies.

Agent	Cancer type	Test description	Sensitivity (95% CI)	Specificity (95% CI)	PPV (95% CI)	NPV (95% CI)	Reference
OTL38	Ovarian	Any lesion (FRα + or -)	83.9%-96.8% *	-	85.3%-92.6% *	-	(34)
		FRα + only	85.9%-97.9% *	-	88.1%-94.9% *	-	
	Lung	>1 cm nodules	95.6% (87.6%–99.1%)	42.9% (9.9%–81.6%)	94.2% (85.8%–98.4%)	50% (11.8%–88.2%)	(40)
		<1 cm nodules	100% (78.2%–100%)	50.0% (1.2%–98.7%)	93.8% (69.8%–99.8%)	100% (2.5%–100%)	
	Pituitary adenoma	Nonfunctioning adenoma	75% (51–90%)	100% (60–100%)	100% (75–100%)	62% (43–77%)	(47)
		FRα-overexpressing adenoma	100% (75–100%)	100% (31–100%)	100% (75–100%)	100% (31–100%)	
		Margin detection with high FRα-expression	100%	100%	100%	100%	(46)
		Margin samples	100% (54–100%)	100% (63–100%)	100% (54–100%)	100% (63–100%)	(55)
BBN-IRdye800CW	Glioblastoma	From 42 foci of fluorescent-guided sampling	93.9% (79.8%–99.3%)	100% (66.4%–100%)	100% (88.8%–100%)	81.8% (48.2%–97.7%)	(52)
		From 89 harvested samples	94.4% (85.6%–98.2%)	88.2% (62.2%–97.9%)	–	–	(53)
cRGD-ZW800-1	Colorectal carcinoma; *ex vivo* lymph node detection	0.005 mg/kg, 2-4 h	74%	79%	71%	82%	(54)
		0.015 mg/kg, 2-4 h	100%	69%	6%	100%	
		0.05 mg/kg, 2-4 h	100%	73%	70%	100%	
		0.05 mg/kg, 18 h	100%	87%	33%	100%	

All rows except white are color coded to indicate diagnostic accuracy within the same reference but as classified by the test description column. *Range with or without patient as random effect. PPV, positive predictive value. NPV, negative predictive value. CI, confidence interval.

### Selection Criteria, Search Strategy and Results

For reviewed agents, we applied the inclusion criteria listed below to the best of our ability:

- Tumor selectivity must be afforded by receptor-mediated uptake
- The conjugate must be <10,000 g/mol
- Must not be a nanoparticle
- Contrast must be provided in the NIRF spectrum (emission range: 750-900 nm)
- Must have advanced to clinical trials
- Must be intravenously administered
- References published on or before November 6th, 2020 were considered
- Only papers published in the English language were reviewed
- Final list of agents discussed was produced based on relevance to the present work

#### Methodology and Search Engines

On November, 6th, 2020, we used the clinical trial registry ran by the United States National Institute of Health (NIH), https://clinicaltrials.gov/, to search the following keywords: “cancer, fluorescence, surgery” and found a total of 251 studies. We then manually checked each study to catalog fluorescent agents used. Applying the aforementioned selection criteria, we identified a total of 6 agents: OTL38, BLZ-100, ABY-029, LS301, cRGD-ZW800-1 and BBN-IRdye800CW. We then searched the name identifier of each agent in https://clinicaltrials.gov/ and https://pubmed.ncbi.nlm.nih.gov/ to retrieve published content. We also searched the keyword “cRGD-ZW800-1” in https://www.trialregister.nl/, a Netherlands trial register for clinical studies being conducted in the Netherlands.

## OTL38 (Small Molecule)

**Figure 1:**
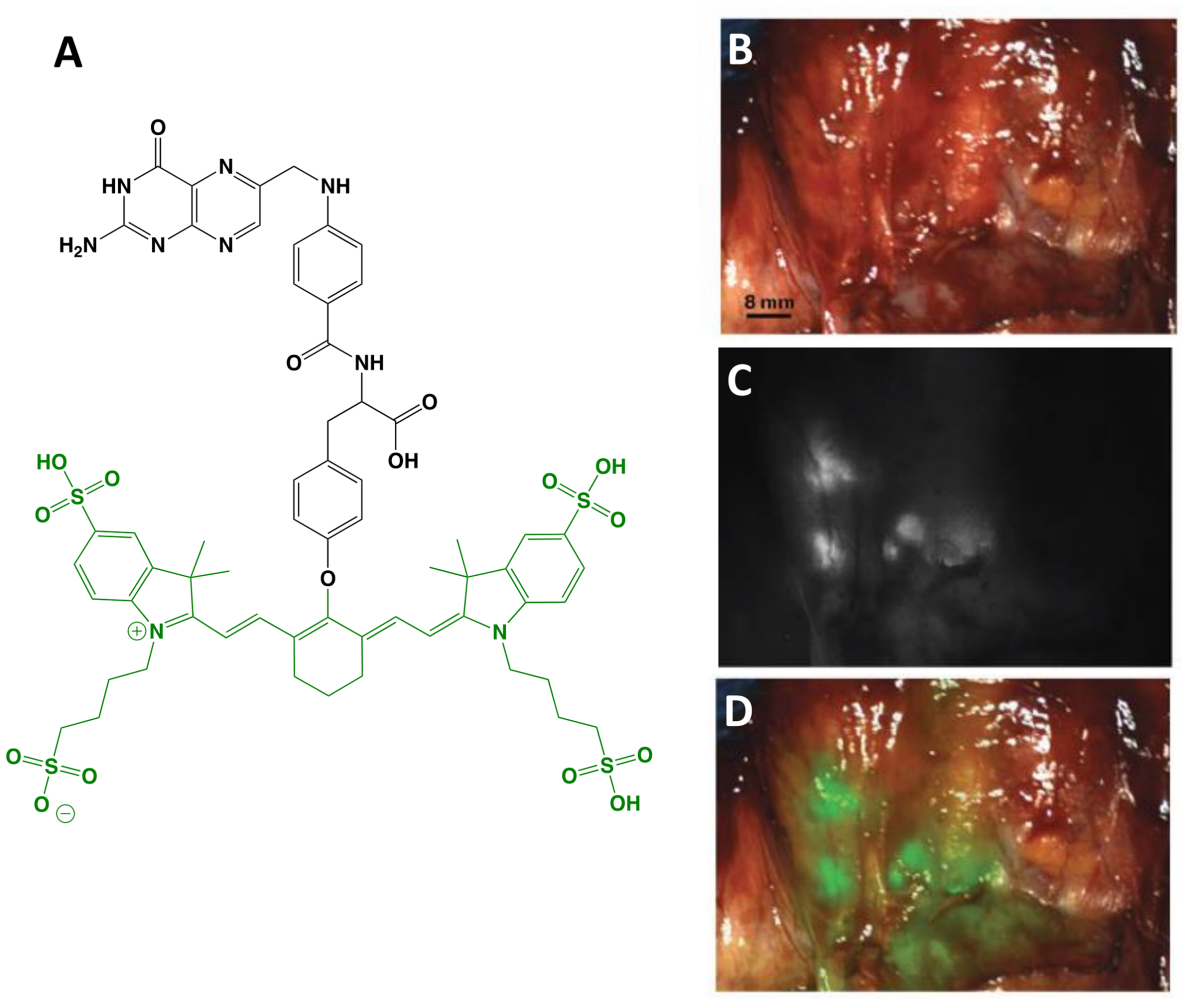
OTL38. Chemical structure; folate motif (ligand) and linker in black, S0456 (dye) in green **(A)**. Intraoperative detection of ovarian cancer metastases with OTL38 administered 2-3 h prior to surgery: white-light only **(B)**, NIRF only **(C)** and white-light with NIRF overlay **(D)**. Panels **(B–D)** are adapted from (30) with permission.

The first clinical report demonstrating that tumor-specific FGS can increase intraoperative detection of cancer used a folate motif linked to fluorescein-isothiocyanate (folate-FITC, EC17; Ex/Em = 494/521 nm) to target the folate receptor α (FRα) in ovarian cancer (29). Unfortunately, the benefits of EC17 are hindered by its emission in the visible wavelength and thus, restricts detection of occult lesions. To overcome detection-depth limits, the folate motif was modified to enable conjugation of the NIRF dye, S0456 (indole cyanine-like green), producing OTL38 (MW = 1,414 g/mol; Ex/Em = 776/793 nm; **Figure 1**) (31). *In vivo* studies using mouse models and canine patients demonstrated the advantages of imaging in the NIRF range and supported the continued development of OTL38 (32, 33). To date, at least 9 clinical trials have begun (Phase I-III) using OTL38 and initial results (**Table 1**) are encouraging as described below.

*Ovarian cancer*: The main role of surgery in ovarian cancer is cytoreduction and thus, the major value of tumor-specific FGS in this setting is to increase debulking rates. Using OTL38, Hoogstins et al. demonstrated an increase of 29% more lesions resected compared to direct visual inspection and palpation (30). To select the optimal dose and time window for imaging, the authors first performed a randomized, placebo-controlled dose-escalation study in healthy volunteers to assess PK in skin and plasma. In patient studies, tolerability, PK, and efficacy were investigated. Efficacy in the intraoperative setting was assessed by measuring (i) TBRs, (ii) congruence between pathology and fluorescence, (iii) number and location of FRα+ lesions identified using standard-of-care and imaging, and (iv) the surgeon’s evaluation of the practicality of the approach. In addition to standard-of-care, study design permitted tumor tissues identified by imaging to be eligible for resection if deemed clinically beneficial and surgically feasible. In another study, which supported progression to Phase III, Randall et al. further investigated safety along with the diagnostic accuracy of OTL38 in 44 patients (34). Results showed the approach to be safe with sensitivity and positive predictive values (PPVs) of over 94.9% when lesions were FRα+ and the statistical model used patients as random effect (**Table 2**).

*Lung cancer*: The major value of tumor-specific FGS in lung cancer is to facilitate tumor localization and afford margin assessment. Several feasibility studies (35–38) using OTL38 in lung cancer have demonstrated accurate identification of known lesions as determined by standard-of-care methods and importantly, enabled detection of otherwise undetectable synchronous subcentimeter processes. Using multivariate analysis, Predina et al. (39) investigated the variables impacting *in situ* tumor fluorescence and found that only nodule depth, but not nodule size, dose administered, imaging time, nor standardized uptake value by positron emission tomography (PET) with fluorodeoxyglucose (^18^F-FDG), predicted *in situ* fluorescence. In another study, Predina and colleagues (40) demonstrated how FGS can synergize with preoperative imaging to translate diagnostic findings into the operating room. Patients who had undergone PET with ^18^F-FDG were eligible for FGS with OTL38. Results showed that 56 of 59 nodules (94.9%) identified preoperatively were targeted by OTL38; remarkably, OTL38 detected 9 additional unknown lesions, which resulted in upstaging and improved management in 12% and 30% of patients, respectively. The authors concluded that the combination of PET (73% sensitivity, 89.3% PPV) with FGS (95.6% sensitivity, 94.2% PPV; **Table 2**) provided superior oncologic outcomes.

*Renal cancer*: Maximal tumor resection without excessive removal of normal parenchyma is the primary role of surgery in renal cancer and FGS could improve outcomes. Folate receptors are highly abundant in normal kidneys, but physiological expression is downregulated upon malignant transformation. Shum et al. (41) and Bahler et al. (42) exploited this phenomenon by applying “reverse-FGS” using OTL38 during surgical resection of kidney tumors. The investigators hypothesized that the lack of FR-mediated fluorescence demarcates renal tumors. Preliminary results in 3 patients supported the hypothesis: “dark” tumors were surrounded by fluorescent parenchyma prior to resection and a uniformly fluorescent parenchyma post-resection indicated intact margins. These observations were confirmed by immunohistochemistry (IHC) analysis.

*Gastric adenocarcinomas, endometrial carcinoma, osteosarcomas, pituitary adenomas:* The clinical evaluation of OTL38 has been expanded to other cancers and yielded favorable results. Accurate staging of gastric adenocarcinomas is challenging and existing techniques are limited. In a pilot clinical trial, Newton et al. (43) demonstrated the ability of OTL38 to color code tumors with a high TBR (4.1 ± 2.9) in 3/5 patients and concluded that the approach is feasible. In 4 high-risk endometrial cancer patients, Boogerd and colleagues (44) showed the utility of OTL38 to paint tumors *in situ* (3/4 patients) and lymph node metastases (n = 16), including one otherwise undetectable malignant deposit. The authors also noted 17 false-positives in 50 non-metastatic lymph nodes, which was caused by targeting of the FR variant, FRβ, expressed in tumor-associated macrophages. Further utility of OTL38 was described for improving pulmonary metastasectomy in osteosarcoma, which could possibly extend survival or provide cure. During minimally invasive pulmonary resection, Predina et al. (45) reported the first successful use of molecular imaging for osteosarcomas in a patient, which was deemed safe, feasible and useful. Resection of pituitary adenomas is an essential treatment, but 20% of patients relapse. In one study, Lee et al. (46) reported that OTL38 afforded tumor visualization in 15/15 patients with an average TBR of 1.9 ± 0.70 (high FRα, 3.0 ± 0.29; low FRα, 1.6 ± 0.43) and 100% sensitivity/specificity (**Table 2**). In another study, Cho et al. (47) evaluated the benefits of non-specific (ICG) *vs.* tumor-specific (OTL38) FGS compared to standard-of-care in pituitary adenomas. Results showed that standard-of-care MIS of pituitary adenomas had 88% sensitivity and 90% specificity; ICG increased sensitivity to 100%, but had a specificity of 29% for both functioning and non-functioning adenomas. By contrast, tumor-specific FGS was 75% sensitive and 100% specific, but when the analysis was limited to FRα+ adenomas, sensitivity and specificity were both 100% (**Table 2**).

## BLZ-100 (Tozuleristide, Tumor Paint™; Peptide)

**Figure 2:**
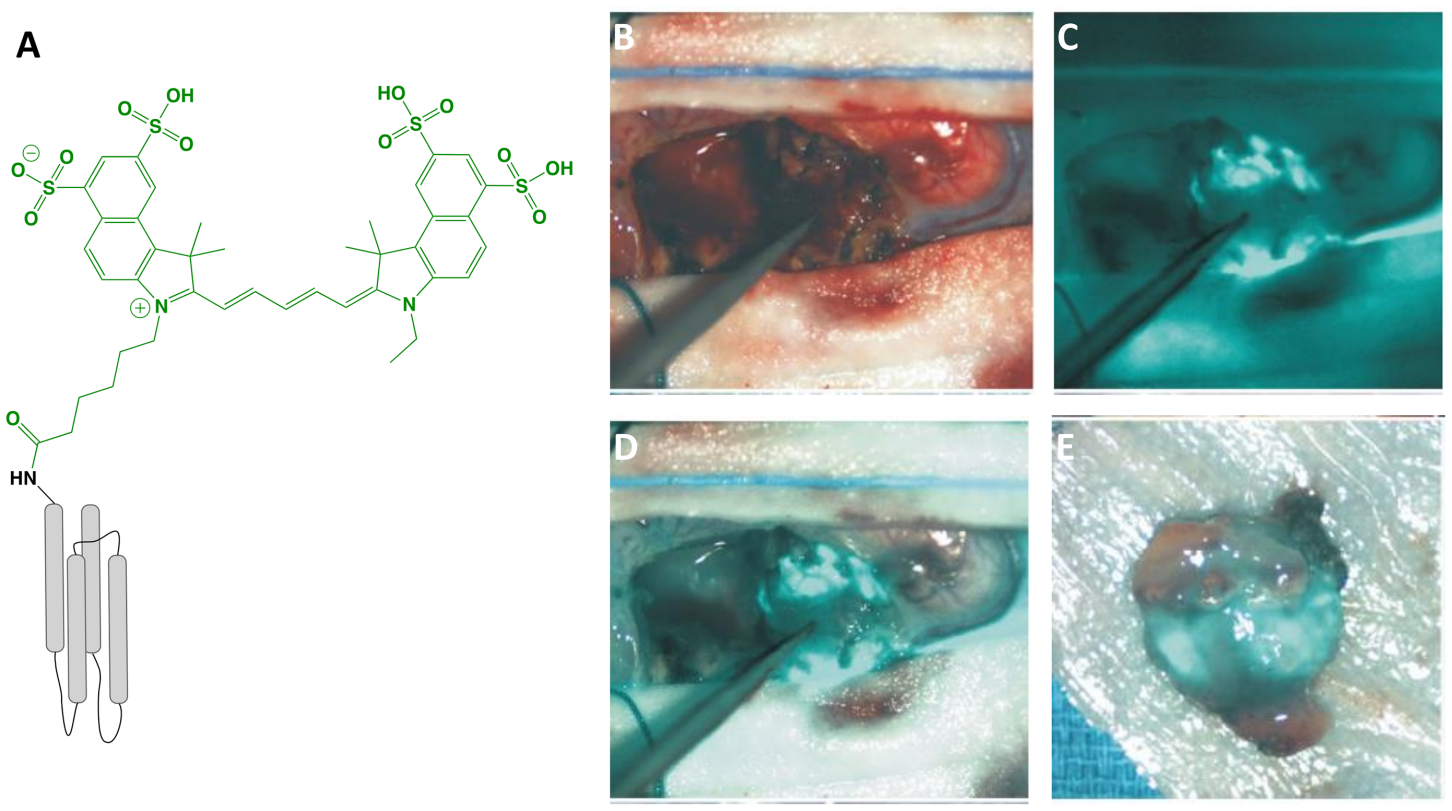
BLZ-100 (Tozuleristide, Tumor Paint™). Schematic* of the peptide CTX (targeting motif; gray) labeled with Cy5.5 (dye; green) **(A)**. Intraoperative glioblastoma imaging with 18 mg total BLZ-100 administered 4 h prior to surgery: white-light only **(B)**, NIRF only **(C)** and white-light with NIRF overlay **(D)**. *Ex vivo* imaging, white-light with NIRF overlay **(E)**. Panels **(B–E)** are adapted from (48) with permission. *Not to scale.

BLZ-100 (MW = 5,124 g/mol; **Figure 2**) is the first receptor-selective NIRF agent for fluorescence-guided neurosurgery that advanced to clinical studies. It is composed of a chlorotoxin (CTX) peptide coupled *via* standard NHS chemistry to the NIRF dye, Cy5.5 (Ex/Em = 675/700-750 nm; ICG derivative). The CTX targeting motif, a 36 amino acid peptide with four disulfide bridges derived from scorpion venom, has been postulated to bind to a number of targets overexpressed in tumors including matrix metalloproteinases, Annexin A2, chloride ion channels, and others (56). The rationale for selecting CTX for FGS drug development came from several studies showing selective targeting of glioma cells compared with non-neoplastic cells or normal brain (57). Furthermore, a radiopharmaceutical analog of CTX, ^131^I-TM-601, demonstrated negligible toxicity in phase I/II clinical trials for human brain cancer therapy (58). In addition to characterization in mouse xenograft models using commercial instrumentation, BLZ-100 has been investigated in combination with a customized device in a drug-device development fashion. Butte and colleagues (59) rationalized their approach on the fact that many commercially available imaging systems are not designed for detection of low ICG concentrations, emphasizing that non-optimal excitation, acquisition and sensitivity settings for imaging a tumor-selective agent may lead to underperformance. From this study, the authors demonstrated an approach to reduce the size and cost of the imaging system, while optimizing sensitivity with low noise. To facilitate clinical translation, the utility of BLZ-100 was evaluated in canine patients using a dose-escalation strategy in combination with commercial and custom imaging devices (60). Additionally, the toxicology and PK profile of BLZ-100 was evaluated in mice, rats, canines, and nonhuman primates, with results supporting first-in-human clinical trials. To date, at least 5 clinical trials have begun (Phase I-III) using BLZ-100 (**Table 1**) and initial results are encouraging:

*Adult and pediatric gliomas*: Patil et al. (48) performed a phase I clinical trial to characterize the safety and utility of BLZ-100 in 17 adults with glioma undergoing surgery. The primary objective was to establish the maximum tolerated dose by conducting a nonrandomized, single-dose, open label, 3 + 3 dose-escalation study (3-30 mg administered 3-29 h before surgery). Secondary objectives included evaluation of PK and fluorescence quantitation of *ex vivo* specimens, whereas exploratory objectives related to the assessment of fluorescence *in situ*. Study design provided standard-of-care to patients, and additionally allowed the surgeon to image the surgical cavity at any point and biopsy fluorescent regions at their discretion. No dose-limiting toxicity was observed and adverse events were not associated with agent administration. Although fluorescence was observed in both low- and high-grade gliomas, signal intensity was dose-dependent and time-independent only for high-grade gliomas. Image analysis was performed by independent reviewers who scored the observed fluorescence as negative, weak (contrast apparent but not well-defined) or strong (well-defined contrast). *Ex vivo* scores yielded 5 negative, 7 weak and 5 strong specimens; *in situ* scores yielded 11 negatives, 4 weak and 2 strong specimens. The investigators attributed the low fluorescence to the unoptimized commercial devices used in the study and intend to couple BLZ-100 with an adequately sensitive imager in the future. The authors concluded that further clinical trials are justified. Lee and colleagues (49) are investigating the safety and utility of BLZ-100 in a phase I study in pediatric brain tumor patients; preliminary results have shown the agent to be safe and yielded fluorescence in 13/15 tumors, including 5/7 low-grade gliomas.

*Breast carcinoma*: BLZ-100 has also been evaluated in breast cancer for fluorescence-guided pathology (50). The objective of this study was to investigate the feasibility of using BLZ-100 to (i) target breast carcinoma and (ii) enable demarcation of surgical margins. 23 patients undergoing surgery received either 6 or 12 mg of agent at least 1 h before the procedure. Resected specimens were imaged using an investigational device and fluorescent patterns were correlated with corresponding hematoxylin & eosin (H&E) stained sections. Furthermore, fluorescent intensity was correlated to clinical pathology, namely grade, histotype, prognostic biomarkers and margin measurements. Results showed that BLZ-100 afforded demarcation of pathologically-confirmed breast carcinoma (low- and high-grade) from normal tissue, independent of molecular marker/hormone receptor status.

## ABY-029 (Affibody)

**Figure 3:**
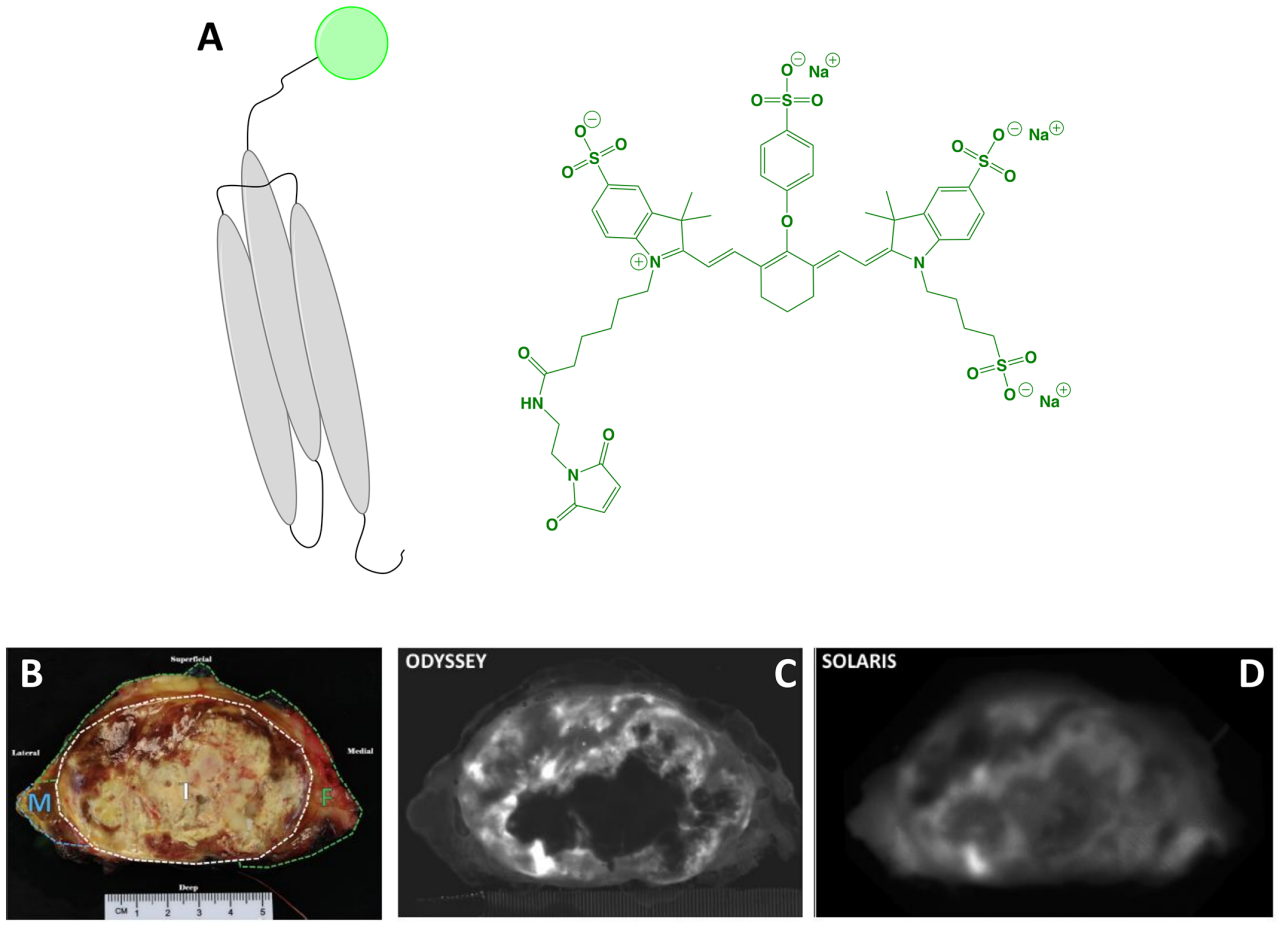
ABY-029. Schematic* of the affibody Z03115-Cys (targeting motif; gray) labeled with IRDye800 (dye, represented as a green circle). The chemical structure of IRDye800 maleimide is also shown **(A)**. *Ex vivo* sarcoma imaging with 237 μg (30 nanomoles) ABY-029 administered 1-3 h prior to surgery: white-light of resected primary (breadloaf) **(B)**, NIRF using a flatbed, closed-box device **(C)** and NIRF using a wide-field device **(D)**. Panels **(B–D)** are adapted from (51) with permission. *Not to scale.

The affibody conjugate ABY-029 (MW = 7,915 g/mol; **Figure 3**) has emerged as an innovative agent to expand the surgical armamentarium targeting EGFR. It is composed of the 58-amino acid synthetic peptide, Z03115-Cys, labeled with IRDye 800CW maleimide (Ex/Em = 774/789 nm). Rigorous evaluation of *in vitro* and *in vivo* performance showed excellent PK, specificity and utility for imaging (28, 61–63). Furthermore, toxicological characterization in Sprague Dawley rats found no pathological evidence of toxicity (64). Elliott et al. (65) demonstrated that ABY-029 is a promising candidate for image guidance of brain tumors, which overexpress EGFR in 50-70% of cases. In orthotopic mouse models of glioma, Elliott and colleagues investigated and compared the image contrast provided by (i) receptor targeting (ABY-029), (ii) metabolic targeting (5-aminolevulinic acid [5-ALA]–induced Protoporphyrin IX [PpIX]; emerging clinical standard for FGS in glioma) and (iii) a permeability tracer (IRDye 680RD). Results showed that receptor targeting outperformed metabolic targeting by increasing TBRs at tumor margins and core by 50% and 60%, respectively. Given that commercial devices may be too insensitive, Elliott et al. (66) investigated the detection limits and *in vivo* efficacy of ABY-029 microdoses in combination with a custom-built imaging module, which enabled superior image contrast at all doses compared to a commercial counterpart. ABY-029 could also provide benefits during resection of soft tissue sarcomas, which overexpress EGFR in 43-78% of cases. To simulate the spectral properties of tissue surrounding sarcoma during wide local excision, Samkoe et al. (67) developed a phantom model to investigate how observed fluorescence signals change in relation to tumor size, tumor depth, bulk tissue type, and imaging system. Results validated the use of subsurface fluorescence to direct the resection of a tumor-mimicking inclusion to a desired margin thickness. ABY-029 has also been investigated for paired-agent imaging. This approach uses a tumor-specific agent in combination with a non-targeted or perfusion agent to subtract nonspecific signals, thereby improving contrast and/or measuring extracellular EGFR regions. Using ABY-029 along with IRDye 680RD conjugated to an affibody control or IRDye 700DX carboxylate, Samkoe et al. (68) and Sardar et al. (69) have demonstrated the feasibility of the approach in preclinical models of soft tissue sarcoma and head and neck cancer.

Notably, the clinical introduction of ABY-029 has followed a Phase 0 approach through submission of an exploratory investigational new drug (IND) to the FDA (70). This trial modality uses the concept of microdosing, namely sub-pharmacologic exposure, which is defined by the FDA as administration of ≤30 nmol of a protein product. It permits analytical toxicology in a single mammalian species and intends to expedite the estimation of key PK parameters of new drugs in a more economically viable pipeline (71). In practice, it is possible to request a modified Phase 0 study to the FDA with doses slightly above a microdose as long as the dose is 100x lower than the no observed adverse effect level in preclinical toxicity studies (72). Understanding that the clinical study of FGS drugs must be designed to maximize tumor contrast, Ribeiro de Souza et al. identified the optimal dose for imaging within a microdose framework in an orthotopic tumor model in rats (72). Results showed that increasing the microdose dose 5-fold, increased signal by 10-fold, which provided a rationale for performing a modified Phase 0 trial. Interestingly, the authors also found that whereas unlabeled cetuximab (anti-EGFR mAb) inhibited ABY-029 binding *in vitro*, it had no effect on *in vivo* tumor contrast when injected 1 or 24 h before ABY-029. To date, at least 3 clinical trials have begun (Phase 0-I) using ABY-029 and preliminary results are encouraging (**Table 1**):

*Soft tissue sarcoma*: Surgery can be curative if complete sarcoma resection is achieved, but positive margin rates of 22-34% have been reported and associated with recurrence. In a proof-of-concept study, Samkoe et al. (51) evaluated the ability of ABY-029 to selectively target EGFR and by extension, provide tumor-specific contrast. A resected specimen from a patient who had been administered a microdose (30 nmol, 237 µg) ~4 h prior to wide local excision was imaged *ex vivo* using flatbed, closed-box and open air, wide-field devices to investigate the observed fluorescence and contrast. Qualitative assessment showed clear fluorescence regardless of device and image analysis indicated comparable signal intensity in the tumor region. Interestingly, the closed-box system had increased image noise, which translated to a reduced contrast-to-noise ratio (CNR), despite having a higher TBR. Analysis of tumor samples for correlation of fluorescence with EGFR IHC was found to be moderately associated (*r* = 0.48), but could be improved by weighting the intensities by the area fraction of EGFR expression. Overall, the authors concluded that these results demonstrate the utility of ABY-029 for selective *ex vivo* imaging of sarcoma.

## BBN-IRdye800CW (Peptide)

**Figure 4:**
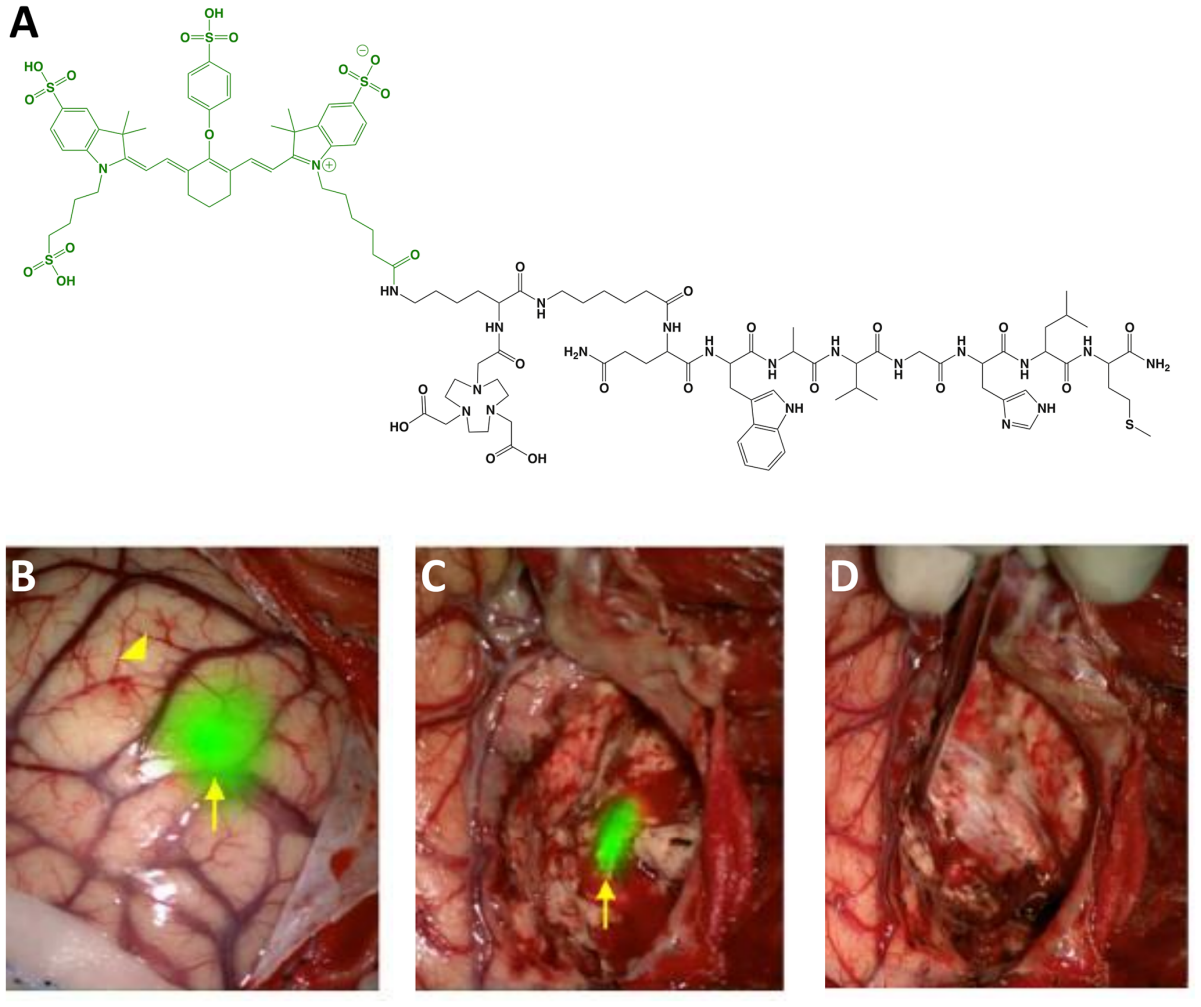
BBN-IRdye800CW. Chemical structure; BBN(7-14) (ligand) and NOTA (Chelator) in black, IRdye800CW (dye) in green **(A)**. Intraoperative glioblastoma multiforme imaging with 1 mg total BBN-IRdye800CW administered 16 h prior to surgery: NIRF prior to resection **(B)**, residual fluorescence after initial tumor removal **(C)** and tumor-cavity after complete resection **(D)**. Panels **(B–D)** are adapted from (53) with permission.

BBN-IRdye800CW (MW = 2,450 g/mol; Ex/Em = 778/795 nm; **Figure 4**) is the first LMW agent derived from a PET radiotracer to advance to clinical studies. It is composed of a gastrin-releasing peptide receptor (GRPR) targeting moiety, BBN(7-14) (amino acid sequence: Gln-Trp-Ala-Val-Gly-His-Leu-Met-NH_2_), linked to the radiometal chelator, NOTA, and NIRF dye, IRdye 800CW NHS ester. The rationale for selecting a clinical radiotracer for FGS drug development is significant since the approach expands the imaging utility of a clinically-validated tumor-selective agent, thereby reducing concerns associated with toxicity and diagnostic efficacy (73). The safety and biodistribution of the parent compound, ^68^Ga-NOTA-Aca-BBN(7–14), was shown to be well tolerated and have favorable PK (74). Preclinical *in vivo* evaluation was performed in an orthotopic model of glioblastoma in mice with a customized imaging device for real time visualization (52). To date, at least 2 clinical trials have begun (Phase I) using BBN-IRdye800CW and initial results are encouraging (**Table 1**):

*Glioblastoma*: Maximum safe resection during glioblastoma surgery remains a challenge despite FGS with non-targeted dyes (e.g., fluorescein sodium) and metabolic markers (e.g., 5-ALA). As an alternative, Li et al. (52) evaluated the utility of ^68^Ga-BBN-IRdye800CW to provide receptor-mediated glioblastoma contrast for increased resection and margin assessment in 14 patients. The aim of the trial was to establish the safety and feasibility of PET and NIRF imaging with the same targeting vector (i.e., theranostics). To investigate the potential to translate pre-operative findings into the operating room, the authors performed PET scans in all patients with the parent radiotracer (n = 7), dual labeled counterpart (n = 4) or both (n = 3). Results showed similar tumor uptake and no significant biodistribution differences between radiotracers. For intraoperative imaging, 1 mg of unlabeled BBN-IRdye800CW was injected to patients 2 h before surgery. The fluorescent signal was well-visualized and provided superior ability to differentiate residual tumor from normal brain when compared to intraoperative white-light microscope imaging. Of note, the authors reported that the approach was not optimal for deep-seated tumors as signal-to-background ratios were not adequate during surgery in some instances. In another study, He et al. (53) investigated the extent of resection and survivability using BBN-IRdye800CW in 29 patients. To avoid confounding variability due to inter-surgeon differences, all procedures were performed by the same surgeon. Results showed that complete resection was achieved in ~83% of cases as determined by post-operative magnetic resonance imaging (MRI) scans. Median overall survival (OS) and progression-free survival (PFS) were 23.1 and 14.1 months, respectively, which is higher than values reported with standard-of-care techniques. Diagnostic accuracy for both studies is shown in **Table 2**.

## cRGD-ZW800-1 (Small Molecule)

**Figure 5:**
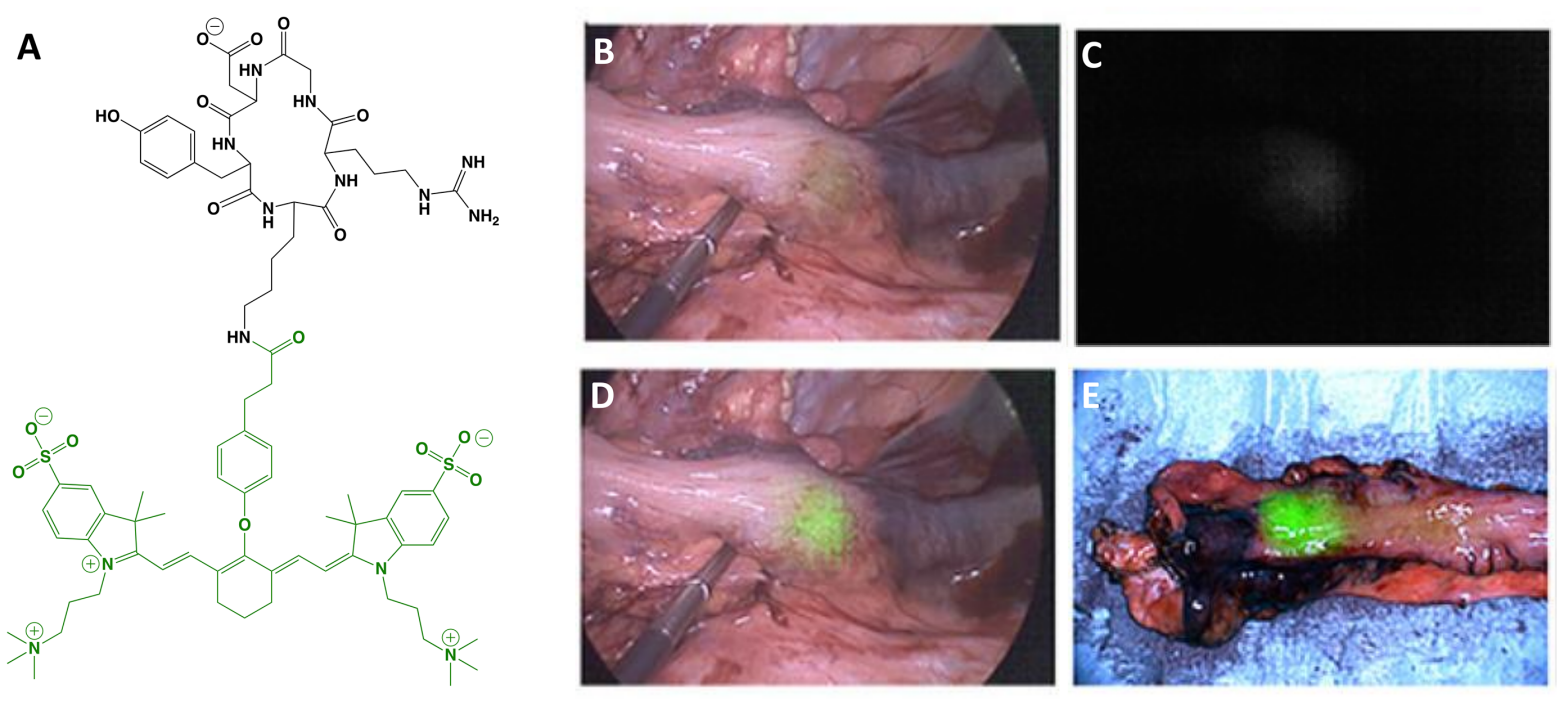
cRGD-ZW800-1. Chemical structure; cRGDyK (ligand) in black, ZW800-1 (dye) in green **(A)**. Intraoperative colon cancer imaging with 0.05 mg/kg cRGD-ZW800-1 administered 18 h prior to surgery: white-light only **(B)**, NIRF only **(C)** and white-light with NIRF overlay **(D)**. *Ex vivo* imaging, white-light with NIRF overlay **(E)**. Panels **(B–E)** are adapted from (54) with permission.

Zwitterionic (i.e., net-neutral) NIRF dyes have demonstrated reduced off-target interactions compared to commonly used NIRF dyes and position as valuable options to increase tumor contrast (75, 76). cRGD-ZW800-1 (MW = 1,729 g/mol; **Figure 5**) is the first tumor-selective agent to incorporate the zwitterionic-dye strategy to advance to clinical studies. It is composed of a cyclic Arg-Gly-Asp peptide, cRGDyK, conjugated to ZW800-1 NHS ester (Ex/Em = 772/788 nm). The cRGD motif targets integrins, which are transmembrane proteins that include α_5_β_1_, α_8_β_1_, α_v_β_1_, α_v_β_3_, α_v_β_5_, α_v_β_6_, α_v_β_8_ and α_II_β_3_. These cell-surface biomarkers have been associated with tumor angiogenesis and migration in a wide range of cancers, making them attractive candidates for tumor-selective FGS drug development. *In vivo* preclinical characterization using a clinical imager showed imaging utility in liver, lung, colorectal, breast, pancreatic and oral orthotopic mouse models, even at microdoses (77–79). In head-to-head comparison with IRDye 800CW and Cy5.5 analogs, cRGD-ZW800-1 demonstrated similar tumor uptake, but superior tumor contrast due to reduced background signal (TBR of 17.2 *vs.* 2.7-5.1) (77). The authors also found that the best scaling factor to extrapolate the dose of a targeted FGS drug from animal models to humans was to adjust by body surface area. Interestingly, a cRGD-ZW800-1 derivative has also been investigated in a dual labeled format, namely cRGD-ZW800-1-Forte-[^89^Zr]Zr-DFO, to potentially develop a companion diagnostic for improved patient selection and surgical planning (80). Nonclinical toxicity studies of cRGD-ZW800-1 in rats were conducted according to ICH M3 (R2), FDA, EMEA, and GLP regulations, and showed no significant clinical signs or pathological changes. To date, at least 2 clinical trials have begun (Phase I-II) using cRGD-ZW800-1 and initial results are promising (**Table 1**):

*Colon carcinoma*: Partial or excessive resection in colon cancer is associated with increased morbidity and mortality. The establishment of neoadjuvant chemoradiotherapy as the standard for locally advanced rectal cancer further complicates eventual surgical resection with MIS due to obscuring of critical visual information. To overcome these challenges, de Valk et al. (54) evaluated the utility of cRGD-ZW800-1 to provide real time discrimination between tumor and normal tissues. The Phase I trial was a randomized, placebo-controlled, double-blinded, microdosing study to evaluate safety, tolerability and PK. Results showed no acute or toxic effects at all doses and measurable blood concentrations of drug up to 8 h after injection. The objective of the Phase II study was to demonstrate the feasibility of the approach and to determine optimal dose and imaging time point. Study design was an open-label ascending dose study in 12 patients and allowed MIS (n = 11) or open surgery (n = 1) 2-18 h after agent administration. Although α_v_β_6_ was selected as the biomarker for IHC staining, selective targeting was solely based by correlating fluorescence and H&E. de Valk and colleagues measured intraoperative TBRs of 1.1-1.6 with fluorescence visualization through the bowel wall being only possible with the highest dose. *Ex vivo* fluorescence intensity in tumor and normal tissues increased in a dose-dependent manner in the 2-4 h post-injection cohort, with comparable TBRs for the median (4.0) and highest (4.1) doses. 18 h post-injection fluorescence intensity at the highest dose decreased in tumor and normal tissue but the TBR (6.2) did not change significantly. Negative predictive value (NPV) and PPV ranges for lymph node detection were 82-100% and 6-71%, respectively, depending on dose and time (**Table 2**). The authors concluded that (i) the approach is feasible but requires further dose and time optimization and (ii) widespread investigation of cRGD-ZW800-1 in cancer surgery is warranted.

## LS301 (Peptide)

**Figure 6:**
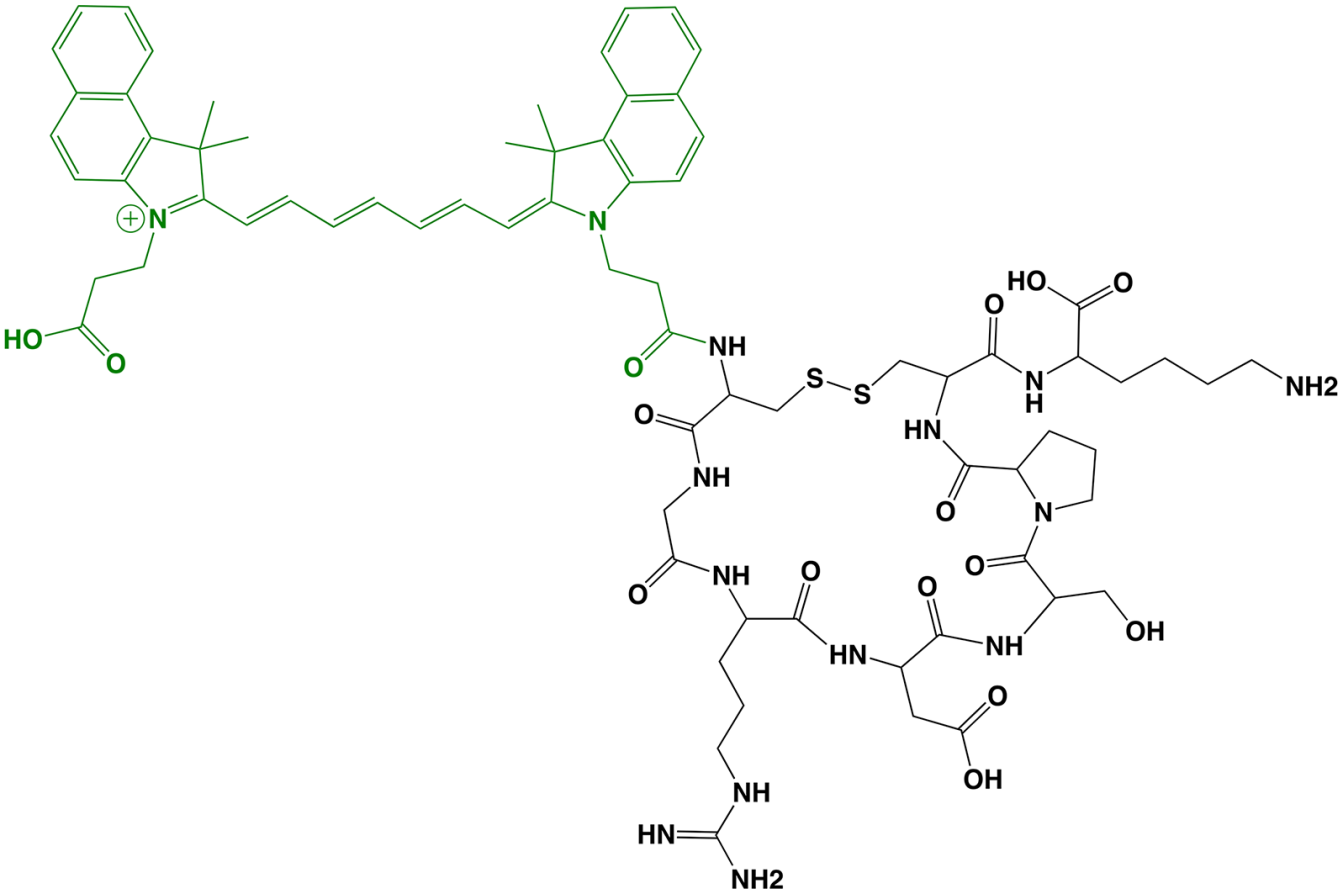
LS301. Chemical structure; c(CGRDSPC)K-OH (ligand) in black, cypate (dye) in green.

Iterative pharmacophore optimization for improved performance is a hallmark facilitated by LMW agents. Such strategy was applied during the development of LS301 (MW = 1,469 g/mol; **Figure 6**) — a tumor-selective FGS agent consisting of the cyclic octapeptide, cyclic (d-Cys-Gly-Arg-Asp-Ser-Pro-Cys)-Lys-OH [c(CGRDSPC)K-OH], conjugated *via* standard methods to the ICG derivative, cypate (Ex/Em = 780/830 nm) (81). The pharmacophore c(CGRDSPC)K-OH was derived from the linear hexapeptide, Gly-Arg-Asp-Ser-Pro-Lys (GRDSPK), to enhance the stability of the first-generation agent, cypate-GRDSPK, *via* intramolecular disulfide cyclization (82). Recently, LS301 has been shown to target the phosphorylated phospholipid-binding protein Annexin A2, which is an abundant post-translational modification of the Annexin A2 preferentially found in tumor microenvironments (82). LS301 is also thought to bind to integrins (e.g., β_3_), but their identities remain unknown (83). Overall, LS301 could be a versatile approach for targeting a wide range of solid tumors. In preclinical *in vivo* studies, LS301 has been shown to selectively accumulate in tumors and metastases of the breast, fibrosarcoma, pancreas and glioblastoma (82). Interestingly, the utility of LS301 for real-time intraoperative imaging has been investigated in combination with “cancer vision goggles” (84), an emerging interface for fluorescence visualization. To date, at least 2 clinical trials have been registered (Phase I-II) using LS301 (**Table 1**).

## Perspective on Emerging Tumor-Selective FGS Technologies

### Significance and Application

FGS holds the promise of color-labeling the surgical field and enhancing surgical outcomes in several ways. Tumor-specific probes that delineate the location and extent of malignant tissues can improve staging accuracy and margin-negative resection; furthermore, they can potentially shorten operative time and avoid excessive removal of normal tissues. Indeed, the anatomy of normal tissues can be distorted by malignant processes, and safeguarding key structures during oncologic surgery can be as important a goal as complete resection of cancer (85). This point is especially important in neurosurgery, where destruction of non-cancerous brain tissue can lead to functional impairments. For gynecologic malignancies, fluorescence is used to evaluate and address disease burden during cytoreductive surgery. Conversely, improved detection of peritoneal dissemination in gastrointestinal cancers, such as cholangiocarcinomas, gastric and pancreatic cancers is critical as accurate tumor detection may spare patients a morbid and futile radical operation. The rapid evolution of MIS, particularly with robotic surgery platforms, has resulted in its widespread application for various cancer types (86–88). FGS technologies can also supplement these emerging surgical techniques with built-in fluorescent imaging systems to overcome the lack of tactile guidance. Maximal benefit, however, can only be realized if the fluorescent signal can accurately define tumor extent.

### Development, Constraints and Outlook

FGS shares many similarities with nuclear medicine (e.g., PET) as a functional imaging modality; however, constraints in the translation of drugs for FGS are unique. For instance, nuclear medicine is inherently quantitative, with well-established methods for determining *in vivo* drug distribution and concentration based on collected images or signals (89). By contrast, the underlying physics of fluorescence imaging in bulk tissue (90), compounded with the myriad of FGS devices (91), leads to uncertainty in the quantitative evaluation of drug performance (92). Particularly, tumor-specific FGS relies on fluorescent drugs that accumulate preferentially in tumors and a device to detect their distribution and concentration to reveal measurable outputs such as tumor contrast (93, 94). For FGS imaging devices, confounding imaging factors include differences in spectral sensitivity, excitation and emission regimes, operating distance, ambient illumination, and efficiency of collection optics (25). Whereas many of these factors could be modeled and accounted for to directly compare commercial devices, this is unlikely to be achieved due to competitive concerns in sharing detailed specifications and optical schematics. Currently, most commercial FGS devices are designed to image ICG, matching its spectral and sensitivity requirements, which may differ from cancer-targeted probes. This is significant because non-optimal excitation and emission collection could lead to underperformance. Imaging efficiency may also be compromised by the tumoral concentration of a targeted dye, as was reported for the BLZ-100 clinical trials. Indeed, the concentration of a targeted dye is expected to be in the nanomolar range, as opposed to the micromolar levels of ICG, and may be a limiting factor (95). Regardless, as seen in **Table 1**, several commercial and experimental NIRF devices are compatible with LMW agents and demonstrate the feasibility of the approach. Optimal image quality [e.g., high signal-to-noise ratio (93)], however, will require instrumentation designed for imaging non-ICG NIRF dyes (e.g., with differing spectra) and with superior sensitivity (e.g., robust low nanomolar detection with low noise); such imaging devices are currently commercially limited or in experimental phases. In response to the growing need for second-generation imagers, specifications of ICG imagers and required capabilities to build upon them were outlined in the seminal work by DSouza et al. (91).

Tumor contrast also depends largely on the differential drug uptake between tumor and normal tissue. This suggests that superior contrast may be achieved by (i) increasing tumor uptake, (ii) decreasing background uptake, or (iii) a combination of (i) and (ii) (92). Points (i-iii) are clinically significant because they could enable contrast detectability with higher certainty, which may directly increase the positive and negative predictive value (96) of tumor-specific FGS for real-time decision making (97). From a chemical structure perspective, a strategy that could advance the specificity of targeted FGS agents involves modulating the physicochemical properties of dyes. Indeed, dye conjugation to tumor-specific vectors can impact the biodistribution of the native ligand, a phenomenon exacerbated for LMW motifs (98). This understanding has, for example, driven the emergence of NIRF dyes with a net-neutral charge (i.e., zwitterionic) that possess reduced nonspecific interactions and faster elimination from normal tissues (75, 76). In combination with high affinity targeting motifs, these attributes could enhance tumor selectivity along contrast in the operating room. Encouragingly, the first clinical application of this technology was with the LMW agent, cRGD-ZW800-1, as previously detailed.

This review focuses on six clinical-stage receptor-targeting LMW FGS agents to not only illustrate the safety and efficacy of the approach, but to also serve as roadmaps. In this sense, knowledge gained from these clinical-stage agents could be applied toward the development of FGS agents that are based on high-value cancer targets across different tumor types (99). For instance, radiolabeled peptides and small molecules have convincingly demonstrated the ability to target the somatostatin receptor subtype-2 (SSTR2) (100) and prostate-specific membrane antigen (PSMA) (101), which are hallmarks of neuroendocrine tumors and prostate cancer, respectively. Surgery is an essential treatment for both diseases and clinical data suggests that incomplete tumor resection can predict recurrence (102, 103). Thus, LMW fluorescent analogs that target SSTR2 (89) and PSMA (104–106) are promising candidates for translation.

Several factors ultimately define the translational route of a FGS agent and include mechanism of targeting, and importantly, whether the dye or ligand has been previously shown to be safe in humans (107). Generally, traditional or exploratory IND enabling studies are conducted first. The drug then undergoes Phase I, II, and III clinical trials to demonstrate safety and efficacy (108), likely resulting in FDA approval of the drug to be device agnostic. There is also the possibility for drug-device combination products to receive FDA approval following a pivotal Phase II clinical trial, which streamlines the regulatory process but requires the drug to be used with the device with which it was paired. Key organizational barriers that may affect the adoption of FGS agents in clinical practice [e.g., evidence-based care, clinical trial design (109)] must also be considered, and could be mitigated through consistent reporting methods and regulatory approaches that allow objective evaluation of these promising technologies [discussed in (94, 107, 108, 110)]. Regardless, the rapid growth of FGS in terms of new drugs, devices, and clinical applications is a testament to the multidisciplinary aspects of the field and its distinct stakeholders.

## Conclusion

Several drug design approaches for tumor-selective FGS have been translated into the clinic with the overarching goal of improving diagnostic accuracy. The physicochemical properties of these drugs play an important role in their *in vivo* performance and can define clinical parameters such as dose and imaging time. FGS mediated by low molecular weight drugs is a safe and potentially efficacious approach to advance the precision of surgical oncology.

## Author Contributions

Conception and design: SHV, CL, AA. Development of methodology: SHV, CL. Acquisition of data: SHV. Analysis and interpretation of data: SHV, CL, HTC, NI, SA, SG, AU, AA. Writing, review, and/or revision of the manuscript: SHV, CL, HTC, NI, SA, SG, AU, AA. Administrative, technical, or material support (i.e., reporting or organizing data, constructing databases): SHV. Study supervision: SHV, CL, AA. All authors contributed to the article and approved the submitted version.

## Disclaimer

The content is solely the responsibility of the authors and does not necessarily represent the official views of the National Institutes of Health.

## Conflict of Interest

CL and AU are employees of OnLume Inc.

The remaining authors declare that the research was conducted in the absence of any commercial or financial relationships that could be construed as a potential conflict of interest.
